# Supplementary material for: MetaQTL: a package of new computational methods for the meta-analysis of QTL mapping experiments
Source: BMC Bioinformatics. 2007 Feb 8;8:49. doi: 10.1186/1471-2105-8-49 (PMC1808479; doi:10.1186/1471-2105-8-49)
Supplement: Additional File 7 — MetaQTL Package : jar file and tutorial. This Zip archive contains both the MetaQTL JAR file and the files of the tutorial. [file 1471-2105-8-49-S7.zip › org.inra.metaqtl/doc/org/metaqtl/main/class-use/CmdLineParser.Option.html]

Uses of Class org.metaqtl.main.CmdLineParser.Option


|  |  |  |
| --- | --- | --- |
| **Overview** \| **Package** \| **Class** \| **Use** \| **Tree** \| **Deprecated** \| **Index** \| **Help** | |  |
| PREV   NEXT | **FRAMES**    **NO FRAMES**     **All Classes** |  |


---


## **Uses of Class org.metaqtl.main.CmdLineParser.Option**

| Packages that use CmdLineParser.Option | |
| --- | --- |
| **org.metaqtl.main** |  |

Uses of CmdLineParser.Option in org.metaqtl.main

| Subclasses of CmdLineParser.Option in org.metaqtl.main | |
| --- | --- |
| `static class` | `CmdLineParser.Option.BooleanOption` |
| `static class` | `CmdLineParser.Option.DoubleOption`             An option that expects a floating-point value |
| `static class` | `CmdLineParser.Option.IntegerOption`             An option that expects an integer value |
| `static class` | `CmdLineParser.Option.LongOption`             An option that expects a long integer value |
| `static class` | `CmdLineParser.Option.StringOption`             An option that expects a string value |

| Methods in org.metaqtl.main that return CmdLineParser.Option | |
| --- | --- |
| `CmdLineParser.Option` | `CmdLineParser.addBooleanOption(char shortForm, java.lang.String longForm)`             Convenience method for adding a boolean option. |
| `CmdLineParser.Option` | `CmdLineParser.addBooleanOption(java.lang.String longForm)`             Convenience method for adding a boolean option. |
| `CmdLineParser.Option` | `CmdLineParser.addDoubleOption(char shortForm, java.lang.String longForm)`             Convenience method for adding a double option. |
| `CmdLineParser.Option` | `CmdLineParser.addDoubleOption(java.lang.String longForm)`             Convenience method for adding a double option. |
| `CmdLineParser.Option` | `CmdLineParser.addIntegerOption(char shortForm, java.lang.String longForm)`             Convenience method for adding an integer option. |
| `CmdLineParser.Option` | `CmdLineParser.addIntegerOption(java.lang.String longForm)`             Convenience method for adding an integer option. |
| `CmdLineParser.Option` | `CmdLineParser.addLongOption(char shortForm, java.lang.String longForm)`             Convenience method for adding a long integer option. |
| `CmdLineParser.Option` | `CmdLineParser.addLongOption(java.lang.String longForm)`             Convenience method for adding a long integer option. |
| `CmdLineParser.Option` | `CmdLineParser.addOption(CmdLineParser.Option opt)`             Add the specified Option to the list of accepted options |
| `CmdLineParser.Option` | `CmdLineParser.addStringOption(char shortForm, java.lang.String longForm)`             Convenience method for adding a string option. |
| `CmdLineParser.Option` | `CmdLineParser.addStringOption(java.lang.String longForm)`             Convenience method for adding a string option. |
| `CmdLineParser.Option` | `CmdLineParser.IllegalOptionValueException.getOption()` |

| Methods in org.metaqtl.main with parameters of type CmdLineParser.Option | |
| --- | --- |
| `CmdLineParser.Option` | `CmdLineParser.addOption(CmdLineParser.Option opt)`             Add the specified Option to the list of accepted options |
| `java.lang.Object` | `CmdLineParser.getOptionValue(CmdLineParser.Option o)`             Equivalent to `getOptionValue(o, null)`. |
| `java.lang.Object` | `CmdLineParser.getOptionValue(CmdLineParser.Option o, java.lang.Object def)` |
| `java.util.Vector` | `CmdLineParser.getOptionValues(CmdLineParser.Option option)` |

Constructors in org.metaqtl.main with parameters of type CmdLineParser.Option

`CmdLineParser.IllegalOptionValueException(CmdLineParser.Option opt,
java.lang.String value)`

---


|  |  |  |
| --- | --- | --- |
| **Overview** \| **Package** \| **Class** \| **Use** \| **Tree** \| **Deprecated** \| **Index** \| **Help** | |  |
| PREV   NEXT | **FRAMES**    **NO FRAMES**     **All Classes** |  |


---
